# Impact of prehospital extracorporeal cardiopulmonary resuscitation for out-of-hospital cardiac arrest on survival with good neurological function: a systematic review and meta-analysis^☆^

**DOI:** 10.1016/j.resplu.2025.100974

**Published:** 2025-05-08

**Authors:** Lawrence Leroux, Nathaniel B. Dennis-Benford, Amy Bergeron, Lionel Lamhaut, Alexis Cournoyer, Brian Grunau, Yiorgos Alexandros Cavayas

**Affiliations:** a Centre de Recherche de l’Hôpital du Sacré-Cœur de Montréal, Montréal, Québec, Canada; b Department of Anesthesiology, Faculty of Medicine, Université de Montréal, Canada; c Department of Anesthesiology, Hôpital du Sacré-Cœur de Montréal, Canada; d University of Virginia School of Medicine, Charlottesville, VA, USA; e Health Library, Université de Montréal, Québec, Canada; f SAMU de Paris and Intensive Care Unit, Necker University Hospital, Assistance Publique-Hôpitaux de Paris (APHP), Paris 75015, France; g Paris Sudden Death Expertise Center, Paris Cardiovascular Research Center (PARCC), INSERM Unit 970, Paris 75015, France; h Paris Cité University, Paris, France; i Department of Family Medicine and Emergency Medicine, Faculty of Medicine, Université de Montréal, Canada; j Department of Emergency Medicine, Hôpital du Sacré-Cœur de Montréal, CIUSSS-NIM, Montréal, Québec, Canada; k Corporation d’Urgences-santé, Montréal, Québec, Canada; l Department of Emergency Medicine, University of British Columbia, Vancouver, British Columbia, Canada; m Centre for Advancing Health Outcomes, Providence Healthcare Research Institute, Vancouver, British Columbia, Canada; n St. Paul’s Hospital, Providence Health Care, Vancouver, British Columbia, Canada; o BC Emergency Medicine Network, Vancouver, British Columbia, Canada; p Department of Medicine, Faculty of Medicine, Université de Montréal, Canada; q Department of Medicine, Critical Care Division, Hôpital du Sacré-Cœur de Montréal, Canada

**Keywords:** Out-of-Hospital Cardiac Arrest, Prehospital Extracorporeal Cardiopulmonary Resuscitation, Extracorporeal Cardiopulmonary Resuscitation, Systematic Review, Meta-Analysis, Survival With Good Neurological Function

## Abstract

**Aim:**

Prehospital extracorporeal cardiopulmonary resuscitation (ECPR) has been proposed to reduce delays in ECPR delivery in refractory out-of-hospital cardiac arrests (OHCA) and improve outcomes. Our aim was to synthesize the literature on outcomes of prehospital ECPR in OHCA, focusing on low-flow times (emergency call to extracorporeal blood flow) and survival with good neurological function, comparing them to in-hospital ECPR when possible.

**Methods:**

We conducted a systematic review and *meta*-analysis of studies reporting outcomes in adult OHCA patients treated with prehospital ECPR. Searches spanned seven databases and relevant grey literature (last updated January 21, 2025). Eligible studies included ≥ 5 patients. The primary outcome was survival with good neurological function (CPC 1–2). Pooled estimates were calculated using random-effects models. Meta-regression assessed the association between low-flow time and survival. Comparative analyses with in-hospital ECPR were performed when possible.

**Results:**

Eight cohort studies involving 305 patients (84% male, mean age 57) were included. Survival with good neurological function was 25% (95%CI: 17–35%). Mean low-flow time was 59 min (95%CI: 46–72). Meta-regression showed a significant inverse association between low-flow time and good neurological outcomes (β = –0.0271, 95%CI: –0.0536 to –0.0006; p = 0.045). Compared to in-hospital ECPR, prehospital ECPR showed no significant difference in survival (RR 1.23, 95%CI: 0.35–4.38) but was associated with significantly shorter low-flow times (mean difference –30 min, 95%CI: –44 to –16).

**Conclusion:**

Prehospital ECPR is associated with a 25% rate of survival with good neurological function. Shorter low-flow times were associated with improved outcomes.

## Highlights

• Prehospital ECPR could improve OHCA survival compared to standard resuscitation.
• Meta-analysis shows 25% survival with CPC 1–2 in prehospital ECPR patients.
• Meta-analysis shows a pooled low-flow time of 58.7 min.
• Shorter low-flow times appear to correlate with increased survival.
• RCTs comparing prehospital to in-hospital ECPR are needed to evaluate the optimal way to deliver ECPR in patients with OHCA.

## Introduction

Out-of-hospital cardiac arrest (OHCA) affects hundreds of thousands annually across North America and Europe, imposing a substantial burden on patients, families, and healthcare systems.^1^, ^2^, ^3^ Survival remains poor, averaging 9% and dropping to 4% in some communities.^1^, ^4^ Moreover, many survivors experience long-term neurologic impairments, compounding the emotional and financial toll.^5^, ^6^.

While many OHCA cases are initially resuscitated with high-quality on-scene care, a significant proportion remain refractory despite optimal efforts.^7^, ^8^ For these patients, extracorporeal cardiopulmonary resuscitation (ECPR), mechanical circulatory support via extracorporeal circulation, may be the only remaining option. The American Heart Association (AHA) now recommends considering the use of ECPR in patients with refractory cardiac arrest when equipment and trained staff are available (Class 2a).^9^ This highlights ECPR’s role in advancing cardiac arrest management, especially after prolonged resuscitation.^10^

ECPR delivery remains logistically complex and highly time dependent. Survival decreases significantly when low-flow time, the interval from arrest to extracorporeal flow, exceeds 70 min.^11^ Transport and extrication may degrade resuscitation quality and reduce the chances of return of spontaneous circulation (ROSC).^12^, ^13^, ^14^ Evidence suggests that initiating hospital transfer around 16 min after professional resuscitation begins may optimize outcomes.^12^ However, even in programs aimed at early transport, departure from the scene typically takes at least 20 to 25 min. 15, 16 Additional delays due to transport leave minimal time for cannulation, limiting the proportion of patients who can benefit from in-hospital ECPR.^17^ For this reason, some regions have decided to explore initiating ECPR in the field.^18^, ^19^, ^20^, ^21^

Prehospital ECPR aims to eliminate transport delays, expanding access to ECPR within the critical 60-minute window.^17^ One study reported that only 37% of refractory OHCA cases can be transported to an ECPR center within a 60-minute window in an urban setting.^17^ In contrast, simulation data suggests that a prehospital ECPR model could reach up to 90% of refractory OHCA in the same window.^17^ Such a system could also contribute to a reduction in low-flow time, with the potential to increase survival.^10^, ^17^

Despite growing interest, the clinical impact of prehospital ECPR remains uncertain. This review aims to synthesize the available literature on prehospital ECPR outcomes, particularly survival with good neurological function and low-flow times, and to compare them to in-hospital models where possible.

## Methods

### Protocol and registration

The protocol was designed in accordance with the Preferred Reporting Items for Systematic Reviews and Meta-Analysis Protocols (PRISMA-P)^22^ and preregistered on the Prospective Register of Systematic Reviews (CRD42024538370) (Supplementary material).

### Clinical question and PICO structure

We aimed to address the following question: In adult patients with out-of-hospital cardiac arrest (Population), does prehospital ECPR (Intervention), compared to in-hospital ECPR (Comparison), improve survival with good neurological function (Outcome)?

### Search strategies

The search strategies were designed by an experienced medical librarian (AB) to retrieve records of studies on prehospital ECPR for OHCA. The preliminary search strategy for Ovid Medline was reviewed by the authors and peer reviewed by another librarian prior to execution using the PRESS Checklist^23^, as recommended by the Cochrane Collaboration’s guidelines on the conduct of systematic reviews.^24^ This primary strategy was then adapted for the other databases using database-specific controlled vocabulary terms and platform-appropriate syntax (Appendix A).

### Searches

The following electronic databases were searched July 3, 2024: Embase (Ovid), Medline ALL (Ovid), CINAHL Complete (EBSCOhost), and the Web of Science Core Collection (Clarivate). Following the execution of the database searches, a grey literature search was developed by AB and validated with the team. AB conducted the grey literature searches on July 12, 2024, including the following sources: the Cochrane Central Register of Controlled Trials (Ovid), the World Health Organization’s International Clinical Trials Registry Platform, and the proceedings of select conferences. Cited references as well as citing references of all final inclusions were exported using Web of Science and screened to identify additional studies. A search update was run on January 21, 2025 across all databases to retrieve any articles published since the original run date. The final, reproducible searches for all databases and grey literature sources are available via Borealis, a Canadian Dataverse Repository (https://doi.org/10.5683/SP3/6OAEWB) in conformity with PRISMA-S.^22^

### Study inclusion and exclusion criteria

We considered studies including adult patients (aged 18 years or more) with OHCA treated with prehospital ECPR. We excluded studies that did not report our primary outcome (survival with good neurological function), or studies in which the primary outcome, specific to prehospital ECPR patients, could not be clearly identified or extracted from the data. Study designs eligible for inclusion were randomized control trials, case-control studies, cohort studies, and case series (of at least 5 patients). Case reports were excluded from our review. We included peer-reviewed studies published in full text or abstracts. We did not include unpublished data. No language restriction was applied. Studies containing patients already analyzed in subsequent publications were excluded.

### Study selection and data extraction process

Duplicates were removed using the deduplication feature of Covidence, and manually for unstructured grey literature results. After removal of duplicates, titles and abstracts from all retrieved records were independently screened by two reviewers (LL and NBDB) using Covidence web-based platform for systematic reviews (Veritas Health Innovation, Melbourne, Australia). Full texts of potentially eligible records were retrieved. Final study inclusion was determined after independent assessment of full texts by two reviewers. Reasons for exclusion were recorded. Any conflicts were resolved by a third reviewer (YAC). Data extraction followed a similar scheme. No automation tools were used. Efforts were made to contact study investigators for missing data or dataset overlap confirmation when necessary.

### Definitions of outcomes and other variables of interest

The primary outcome of interest was survival with good neurological function, defined as Cerebral Performance Category (CPC) 1 (good cerebral performance) or 2 (moderate cerebral disability). In addition to CPC, we also checked for the use of other validated neurological outcome scales, including the modified Rankin Scale (mRS) and the Extended Glasgow Outcome Scale (GOS-E). The primary explanatory variable of interest was the low-flow time (defined as the time between the emergency call and ECMO initiation). Additional data extracted included patient characteristics such as age, sex, and cause of cardiac arrest.

### Quality assessment

Risk of bias was assessed using two tools, depending on study design. For single-arm, non-comparative case series, we used the NIH Quality Assessment Tool for Case Series Studies (Appendix B), as it provides a pragmatic framework suited to descriptive designs. For the subset of comparative cohort studies that evaluated in-hospital versus prehospital ECPR outcomes, we applied the ROBINS-I tool, as recommended by the Cochrane Handbook for non-randomized comparative studies. Additionally, we performed a GRADE assessment of the certainty of evidence for the primary outcome and primary explanatory variable in comparative studies. Two independent reviewers (LL, NBDB) conducted all assessments, with any discrepancies resolved by a third reviewer (YAC). No automation tools were used in the risk of bias or certainty assessments.

### Data synthesis and analysis

We planned to *meta*-analyze the primary outcome (proportion of patients with survival with CPC 1–2) and primary explanatory variable (low-flow time) in the patients treated with prehospital ECPR. If there were any comparative effectiveness study (i.e., including patients treated with in-hospital ECPR), we planned to *meta*-analyze the data to estimate the effect of prehospital ECPR in comparison to in-hospital ECPR initiation. Data preparation included calculating means and standard deviations when not reported, as described by Wan et al.^25^ Random-effects model were used at all times to account for heterogeneity in settings. Heterogeneity was assessed using the I^2^ statistic and τ^2^ estimates.

For survival with good neurological function, we used a random-effects model to estimate the pooled proportion of patients achieving survival with CPC 1–2, reported with corresponding 95% confidence intervals (CIs). For low-flow time, we used the same random-effects model to estimate the pooled weighted mean and corresponding 95% confidence intervals (CIs) across included studies. Forest plots were used to visually display individual study results and pooled estimates, and a random-effects model was employed to account for heterogeneity. Heterogeneity was assessed using the I^2^ statistic and τ^2^ estimates. Although the number of studies was fewer than 10, we conducted *meta*-regression in an exploratory, hypothesis-generating context.

In the subset of studies that allowed direct comparison between prehospital and in-hospital ECPR, we conducted additional *meta*-analyses. We first calculated the risk ratio of survival with good neurologic outcome then calculated the standardized mean difference of low-flow time between prehospital and in-hospital groups.

Analyses were performed using R (version 4.2)^26^ with the meta^27^ and metafor^28^ packages.

## Results

**Fig. 1:**
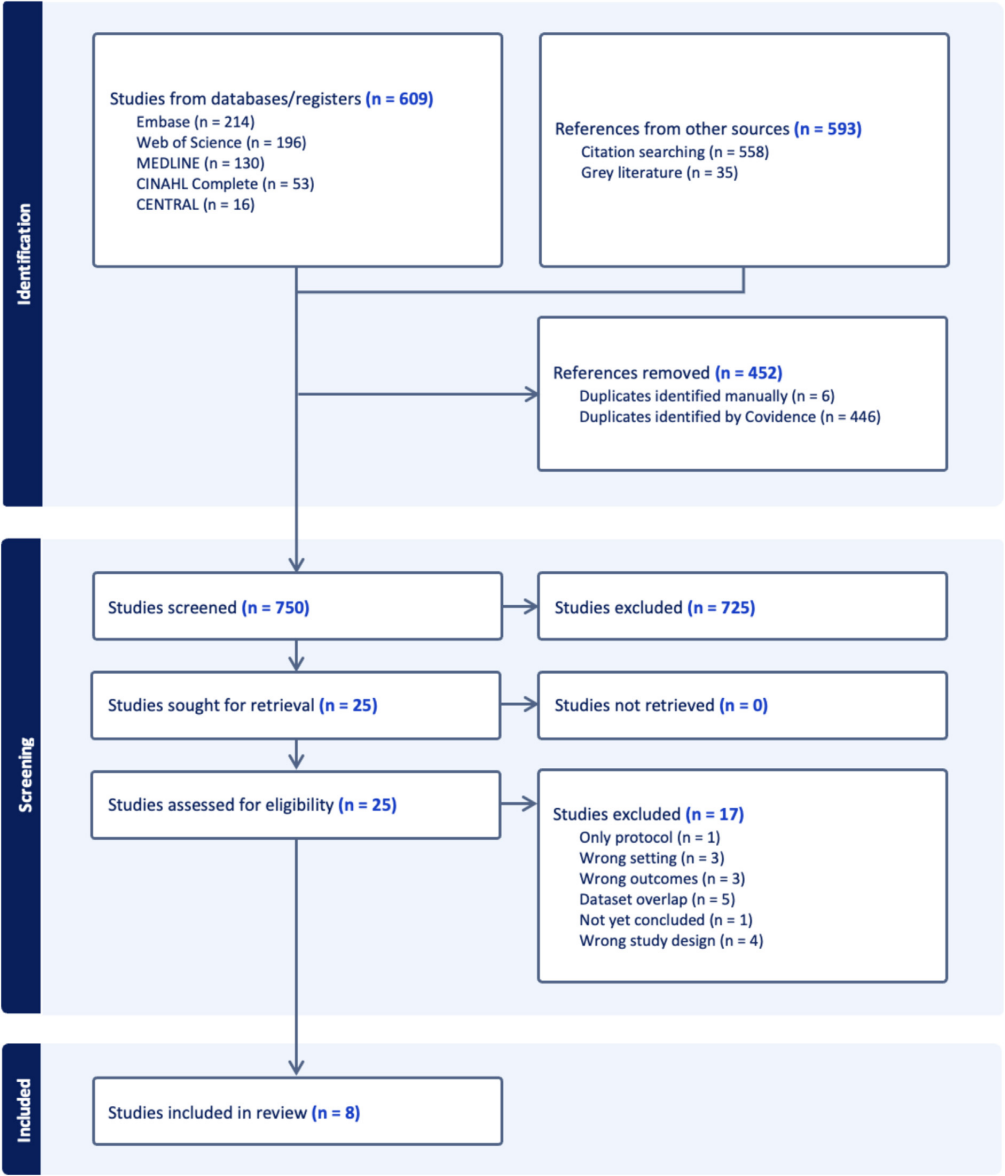
Flow diagram.

A total of 1202 citations were retrieved across all sources and dates. A total of 750 references were left to screen following deduplication: 725 were excluded after abstract screening and 25 full articles were reviewed. As part of the grey literature and trial registry search, we identified three ongoing studies evaluating prehospital ECPR: the ON-SCENE trial (NCT04620070), a stepped-wedge cluster randomized trial; the RACE trial (NCT06789978), a single-center prospective feasibility study in Prague; and the PRE-CARE study (ACTRN12625000318482), another single-center prospective feasibility trial based in Sydney. These studies were excluded from analysis due to unavailability of outcome data. The reasons for studies exclusion are listed in the flow diagram in Fig. 1. Articles by Philipp et al.,^29^ Hutin et al.,^30^ Lamhaut et al.,^31^, ^32^ Bougouin et al.^33^ and Pozzi et al.^34^ were excluded because their population were already incorporated in three more recent articles^35^, ^36^, ^37^, which were prioritized due to their larger sample sizes. While CPC 1–2 was used as the primary neurological outcome, we also screened for studies using mRS or GOS-E, and no studies were excluded solely based on use of a different neurological outcome scale.

### Study characteristics

The eight selected studies included a total of 305 prehospital ECPR cases. The studies were retrospective/observational (n = 6) and prospective/feasibility (n = 2) non-controlled studies. Other than maximal low-flow times, the inclusion/exclusion criteria were similar between the studies and in line with current guidelines (Appendix C). Overall, 83.9% (95% CI 79.7–88.1) of cases were men, the weighted mean average age was 57 years old (95% CI 56.5–58), 77.0% (95% CI 74.0–80) of cases had a cardiac cause for their cardiac arrest and 63.1% (95% CI 60.4–65.9) had an initial shockable rhythm. (Table 1).

**Table 1:** Selected studies characteristics.

First Author	Country	Design	Sample Size	CPC 1–2N (%)	CPC timing	Low-Flow Time (min ± SD)	Age (years ± SD)	Shockable RhythmN (%)	Other findings
Lamhaut (2017)^35^*	France	Retrospective Case series	46	2 (4,3%)	ICU discharge Or 28 days	83 ± 26,9	51 ± 12,9	26 (56,5%)	Stringent inclusion criteria, early ECPR deployment, and epinephrine restriction (5 mg) increased survival
Petermichl (2021)^42^	Germany	Retrospective Case series	69	17 (24,6%)	Hospital discharge	48 ± 9,3	58 ± 14,1	13 (20,6%)	Biomarkers (S100, NSE, lactate, d-dimers and IL6) predicted neurological outcomes
Pozzi (2022)^36^	France	Retrospective Case series	30	7 (23,3%)	Hospital discharge	71 ± 15,4	58 ± 11,1	21 (70%)	
Soumagnac (2023)^46^	France	Retrospective Case series	17	7 (41,2%)	28 days	N/A	53 ± 15.6	11 (64,7%)	Hypothermic, non-hypoxic, refractory OHCA are associated with good neurological outcomes
Richardson (2023)^43^	Australia	Feasibility/ Pilot Trial (non-controlled)	10	4 (40%)	Hospital discharge	50 ± 8,5	46 ± 10,5	8 (80%)	Prehospital ECPR was feasible using an experienced ECMO team from a single center
Trummer (2024)^39^	Germany	Multicenter Prospective Observational	14	6 (42,9%)	90 days	37 ± 20	59,3 ± 12,7	9 (69,2%)	Controlled automated reperfusion of the whole body has the potential to improve outcomes after prolonged CPR
Singer (2024)^38^	United Kingdom	Feasibility/ Pilot Trial (non-controlled)	5	0 (0%)	Hospital discharge	47 ± 9,8	61 ± 6,3	3 (60%)	Sub-30 min low-flow were not possible in an urban environment
Khoury (2024)^37^	France	Retrospective Case series	114	24 (21,1%)	1 year	76 ± 18,5	64 ± 11,1	97 (85,1%)	Most CPC 1–2 survivors recover normal heart function (NYHA-FC 1) within one year, and half return to work

* Only patients from period 1 were included.

### Risk of bias

**Fig. 2:**
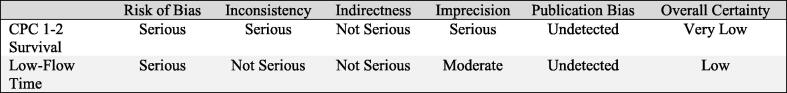
GRADE Summary Table for comparative cohort studies.

**Fig. 3:**
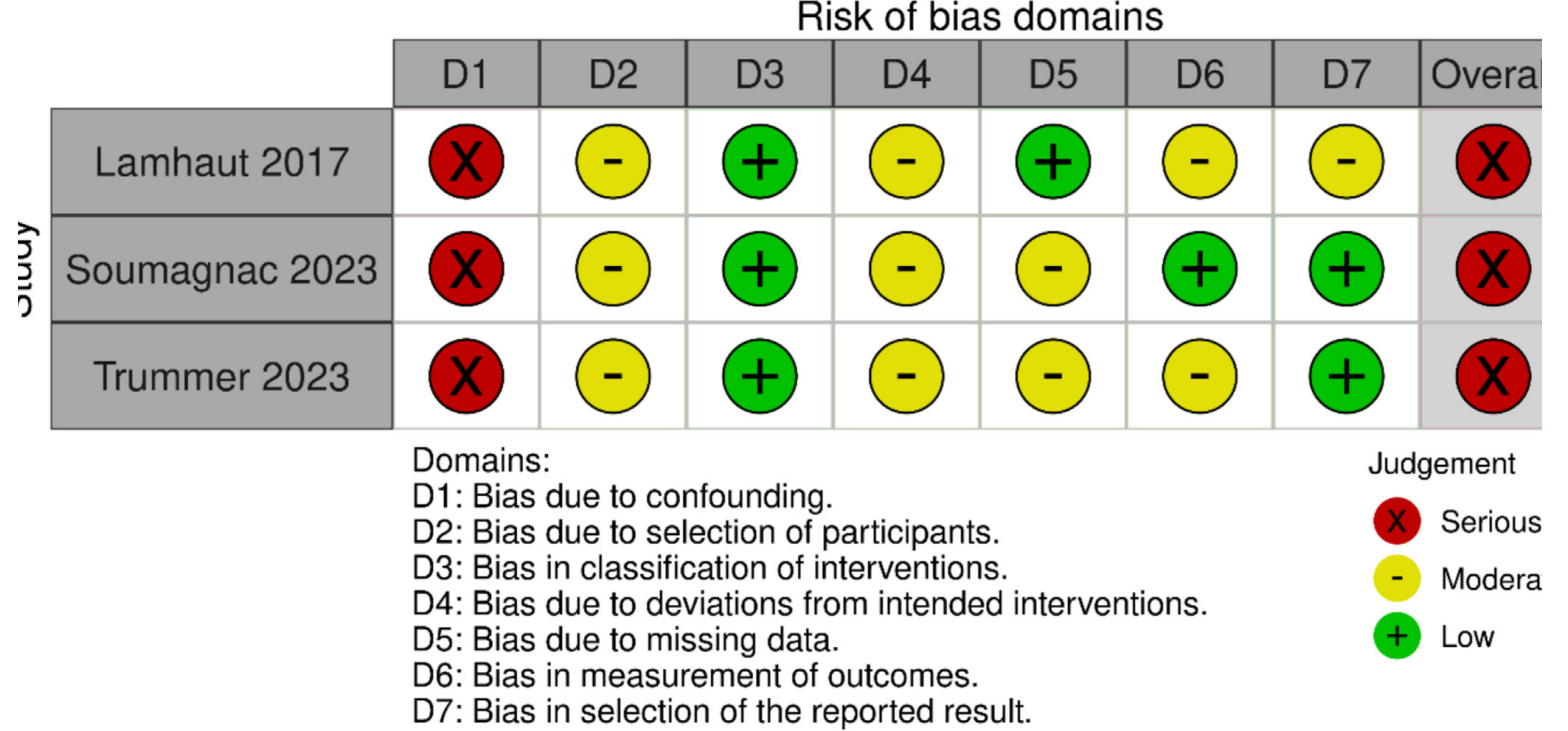
Risk of Bias Assessment for comparative cohort studies using ROBINS-1 tool.

**Fig. 4:**
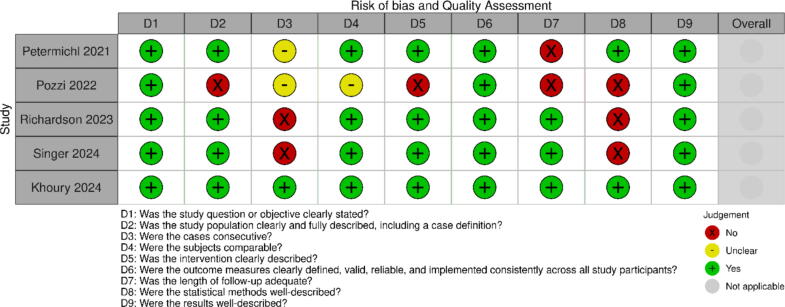
NIH Case Series Quality Assessment tool evaluation of non-comparative case series.

Given the observational and predominantly non-comparative nature of the included studies, the overall certainty of evidence was rated as very low for survival with good neurological function and low for low-flow times, according to the GRADE framework (Fig. 2). The ROBINS-I assessment revealed a serious risk of bias in the three comparative studies due to confounding, selection bias, and lack of adjustment for key variables (Fig. 3). Most of the remaining studies were single-arm case series with variable methodological quality (Fig. 4), as evaluated using the NIH Quality Assessment Tool. Selection bias was common, as patient inclusion was often not consecutive or clearly described. In addition, few studies reported post-ECPR ICU management, limiting assessment of whether differences in post-resuscitation care may have influenced outcomes. Follow-up bias also affected several studies, with CPC 1–2 typically assessed at hospital discharge or one month, without long-term neurological evaluation. This limits our ability to assess sustained neurological recovery. Finally, learning curve bias may have contributed in studies reporting initial clinical experiences, where early outcomes may reflect inexperience rather than the true potential of prehospital ECPR.

### Outcome and explanatory variables

**Fig. 5:**
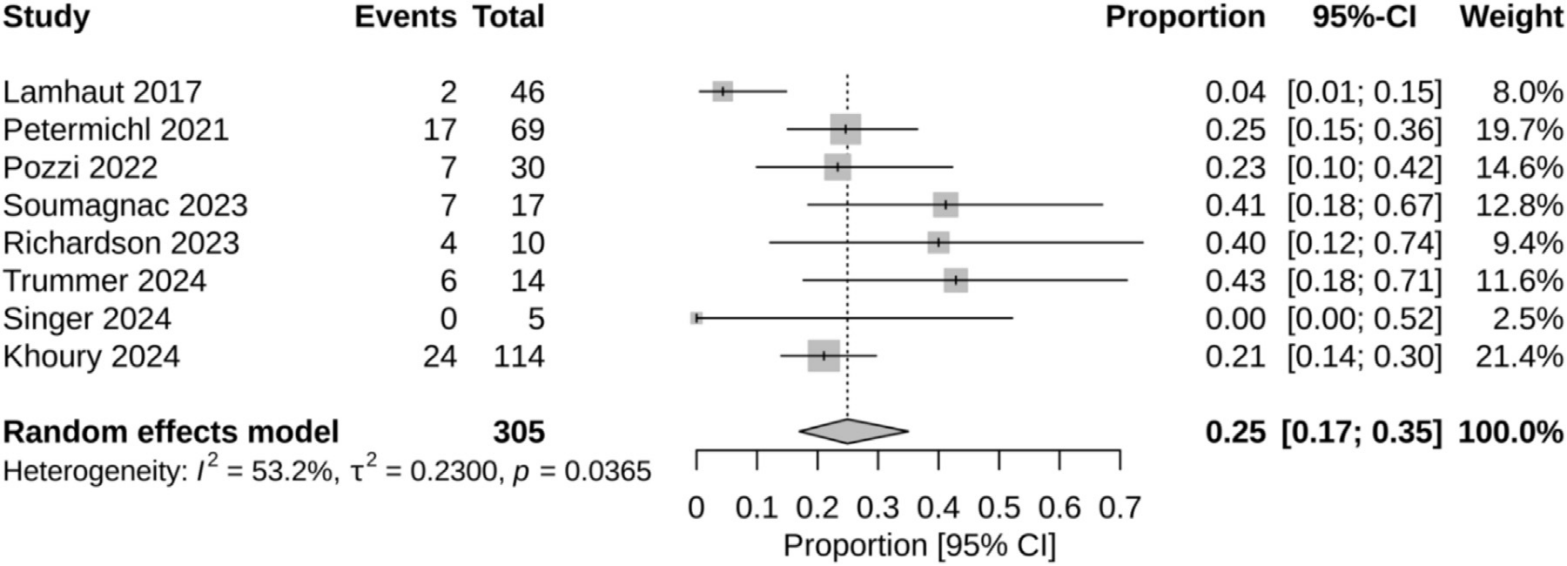
Meta-analysis of proportion survival with good neurological function (CPC 1–2) for prehospital ECPR.

**Fig. 6:**
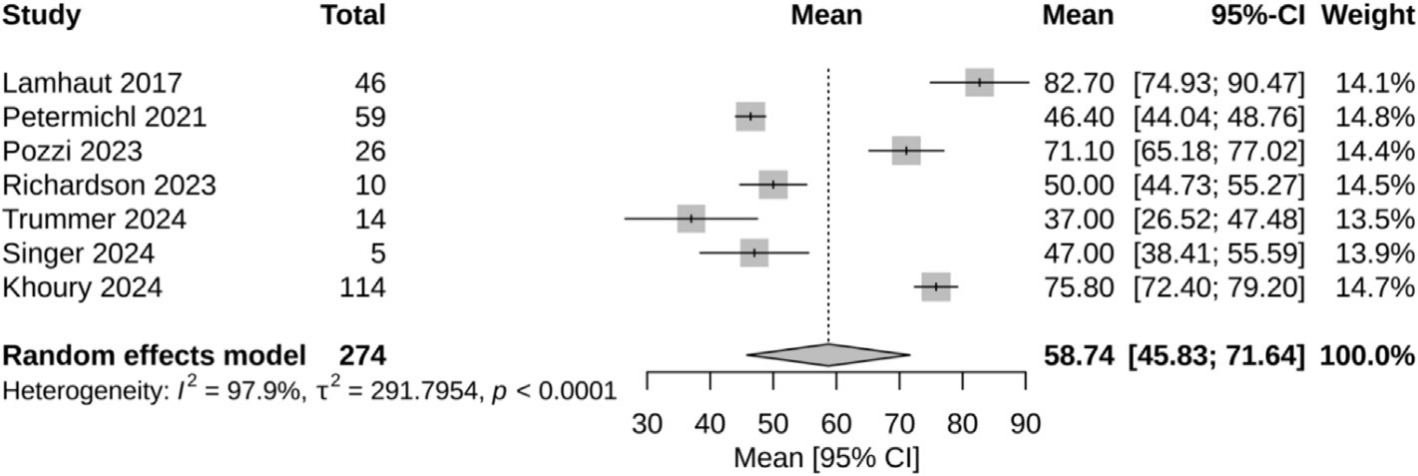
Meta-analysis of low-flow times for prehospital ECPR.

Survival with good neurological function ranged from 0% ^38^ to 43% ^39^ in individual studies. In our *meta*-analysis, the pooled survival rate with good neurological function, estimated using a random-effects model, was 25% (95% CI: 17% − 35%), with high heterogeneity (I^2^ = 53%) (Fig. 5). Low-flow times ranged from 37 min ^39^ to 83 min ^35^. In our *meta*-analysis, the pooled low-flow time of selected studies, estimated using a random-effects model, was 59 min (95% CI: 46 – 72), with critical heterogeneity (I^2^ = 97.9%) (Fig. 6).

**Fig. 7:**
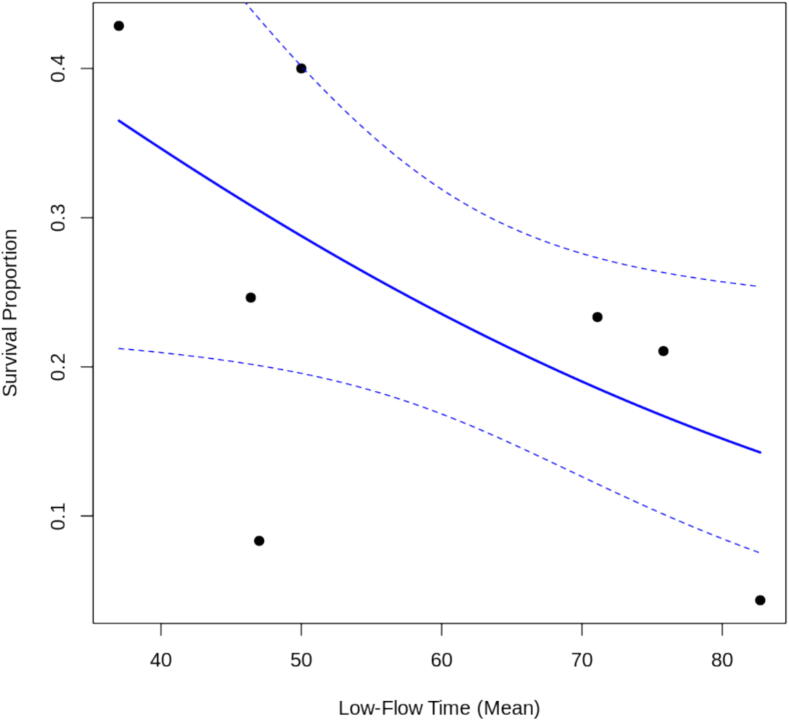
Meta-Regression of survival with good neurological function on low-flow time.

Meta-regression of survival as a function of low-flow time was conducted using the seven studies in which low-flow times could be extracted (Fig. 7). When low-flow time was added as a moderator, the residual heterogeneity dropped (τ^2^ = 0.1029, *I*^2^ *= 30.71%*), indicating that low-flow time explained about 55% of the original heterogeneity. We observed a negative relationship between low-flow time and survival (β= − 0.0271, 95% CI: −0.0536 to − 0.0006, p = 0.0448), suggesting that each additional minute of low-flow is associated with reduced odds of survival.

### Comparison of survival and low-flow times between prehospital and In-Hospital ECPR

**Fig. 8:**
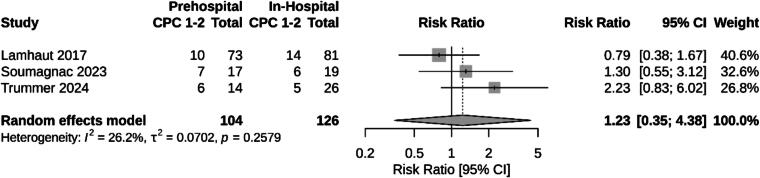
Meta-analysis of survival with good neurological function (CPC 1–2) comparing prehospital and in-hospital ECPR for out-of-hospital cardiac arrest.

Three studies provided comparative data on survival with good neurological function (CPC 1–2) between prehospital and in-hospital ECPR. The pooled risk ratio for survival with CPC 1–2 was 1.23 (95% CI: 0.35–4.38; p = 0.26), indicating no statistically significant difference between the two groups (Fig. 8).

**Fig. 9:**
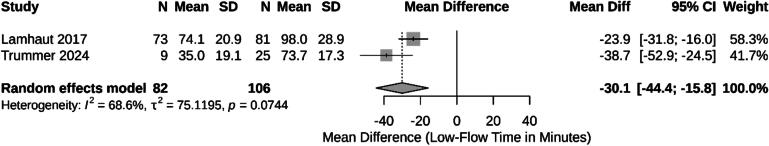
Meta-analysis of low-flow times comparing prehospital and in-hospital ECPR for out-of-hospital cardiac arrest.

Two studies reported comparative data on low-flow times. The pooled mean difference favored prehospital ECPR, with a reduction of 30.1 min compared to in-hospital ECPR (95% CI: –44.4 to –15.8; p < 0.001), representing a statistically significant difference (Fig. 9).

## Discussion

This systematic review identified 13 studies reporting outcomes for patients with OHCA treated with prehospital ECPR. After excluding overlapping datasets, *meta*-analysis of the remaining 8 studies showed a pooled survival with good neurological function (CPC 1–2) of 25% and a pooled low-flow time of 59 min. There was substantial heterogeneity across studies in both patient selection and outcome definitions.

Several limitations must be acknowledged when interpreting these findings. The studies included in this review were small, predominantly retrospective and non-controlled, introducing a high risk of selection and confounding biases. Although outcomes were encouraging, the lack of randomized controlled trials (RCTs) further exacerbates these concerns, preventing definitive conclusions regarding the causal impact of prehospital ECPR. Additionally, significant heterogeneity in patient selection criteria, especially in terms of low-flow time thresholds, may contribute to the observed variability in survival with good neurological outcomes rates.

A consistent trend observed in our analysis is the correlation between low-flow time and survival with good neurological function among ECPR recipients. Although our *meta*-regression supported an inverse association between low-flow time and survival, this analysis included fewer than ten studies and should be interpreted with caution due to the potential for overfitting and limited statistical power. We emphasize that this finding is exploratory and hypothesis-generating, rather than confirmatory. Noteworthy recent studies have similarly suggested that shorter low-flow durations are associated with improved outcomes.^40^, ^41^ Consequently, setting a maximal limit for low-flow time in ECPR candidacy could substantially enhance survival. A 60-minute cut-off from arrest to ECMO flow has been proposed in the literature.^11^ However, implementing such criteria may prove challenging for in-hospital ECPR delivery in OHCA, potentially reducing the number of eligible candidates.

In fact, leaving the scene too early could compromise outcomes. Prior data suggest that intra-arrest transport may be detrimental if performed prior to 15–16 min.^12^, ^14^ This timeframe allows most non-refractory cardiac arrest patients to achieve ROSC before transport, ensuring that only those truly in need of ECPR are considered for escalation. Moreover, patients who remain in cardiac arrest beyond this period are unlikely to achieve ROSC even with prolonged on-scene resuscitation, making further efforts on-site less beneficial.

Considering the time required for EMS arrival, on-scene resuscitation, and patient extraction, the remaining window for hospital transport becomes significantly constrained, further limiting the number of patients who could realistically benefit from in-hospital ECPR. Simulation data suggests that in-hospital ECPR models for OHCA may only reach 40% of potential candidates (given that patients are not considered candidates if low-flow time exceed 60–70 min, thus limiting the geographical footprint), while prehospital models boast a capture rate nearing 90%.^11^

In our review, the pooled low-flow time remained high at 59 min. Studies in which protocols allowed patient inclusion up to 100 min of low-flow time, combined with large sample sizes, likely contributed to relatively high pooled mean.^35^, ^36^, ^37^ Conversely, studies by Singer et al.,^38^ Trummer et al.,^39^ Petermichl et al.,^42^ and Richardson et al.^43^ reported significantly shorter low-flow times, which would be difficult to achieve using an in-hospital ECPR model,^16^, ^44^, ^45^ showcasing the potential reduction in low-flow time associated with prehospital ECPR. Despite these promising figures, the small sample sizes of these studies and the lack of prospective comparative studies between prehospital and other models necessitates cautious interpretation. When analyzing the effect of prehospital ECPR on low-flow time, it is important to consider that the very existence of prehospital ECPR teams may lead to treating patients with prolonged low-flow times who would not have been considered candidates for in-hospital ECPR, thus inflating the observed mean low-flow time in prehospital cohorts. Furthermore, direct comparisons between prehospital and in-hospital low-flow times should take into account differences in geographical coverage and operational perimeters, as these factors influence patient inclusion criteria and transport logistics.

We identified three non-randomized-controlled studies^35^, ^39^, ^46^ comparing outcomes of patients treated with a prehospital vs in-hospital ECPR strategy. The pooled analysis of survival with good neurological function (CPC 1–2) showed no statistically significant difference between prehospital and in-hospital ECPR (RR 1.23, 95% CI 0.35–4.38; p = 0.26). Of note, Lamhaut et al., the largest study included in this comparison, reported relatively long low-flow times in both groups (74 vs. 98 min), which may have contributed to the absence of observed survival differences. Additionally, the Soumagnac et al. study focused exclusively on hypothermic patients, a population in which low-flow time reduction may not necessarily be as beneficial.^46^ Although prospective, the Trummer et al. study compared centers that did not uniformly provide both prehospital and in-hospital ECPR, introducing center-level confounders such as differences in patient selection, team expertise, protocols, and post-resuscitation care. These factors limit internal validity and hinder attribution of observed outcomes to the ECPR strategy alone.

Nevertheless, these findings remain encouraging. The pooled survival rate in our review (25%) compares favorably with published in-hospital ECPR survival rates (18%)^47^ and substantially exceeds the proportion of survival with good neurological function reported with conventional CPR (0.3%) for similar low-flow durations.^41^ Moreover, we found a consistent and statistically significant reduction of low-flow time associated with prehospital ECPR across the studies and in the pooled analysis (mean difference –30.1 min, 95% CI –44.4 to –15.8; p < 0.001). These findings suggest that prehospital ECPR could offer potential benefits, even after prolonged low-flow states. However, given the small sample sizes and methodological heterogeneity, these findings should be interpreted with caution. Larger-scale, prospective, and controlled studies are imperative to establish and validate the comparative effectiveness of prehospital versus in-hospital ECPR and to better inform the selection of optimal resuscitation strategies for out-of-hospital cardiac arrest.

Beyond clinical outcomes, integrating resource-intensive interventions like prehospital ECPR into the chain of survival presents logistical and operational challenges. These programs require highly skilled and experienced dedicated human and material resources that are not easily redeployed to other tasks when not in use, unlike in-hospital ECPR. To improve the cost-effectiveness of prehospital ECPR, further research is needed to determine the optimal deployment model, team configuration, and strategies for resource utilization during periods without active ECPR cases.

Additionally, in non-physician staffed EMS systems like those in North America, prehospital ECPR represents a fundamental shift in human resources to incorporate advanced resuscitation techniques in the field. While this shift may improve access to ECPR, it must not come at the expense of interventions that strengthen the early stages of the chain of survival, such as early cardiac arrest recognition, bystander CPR, first responder interventions, and high-quality paramedic-delivered CPR, which are essential for optimizing survival. In contrast, in countries with established physician-led prehospital systems, implementing prehospital ECPR may require a comparatively lower investment, as core personnel are already in place and additional costs are largely related to training and equipment.

Despite these challenges, prehospital ECPR appears to compare favorably in terms of cost-effectiveness when evaluated against other resuscitation strategies, such as mandatory CPR training programs and public access AED deployment, making it a promising option for improving OHCA outcomes.^48^, ^49^, ^50^ An economic evaluation comparing these strategies and their impact on quality-adjusted life years (QALYs) could further inform decision-making regarding optimal resource allocation.

Organ donation potential is also an important factor in cost-effectiveness analyses. Notably ECPR has demonstrated potential in augmenting organ procurement compared to standard resuscitative efforts^33^, ^51^, ^52^, ^53^ Approximately 20% of ECPR-attempted patients may qualify as organ donors,^51^, ^54^ with notable kidney donation rates.^51^, ^52^, ^54^ Besides, these grafts have also been shown to have similar one-year graft survival compared to other donors.^51^, ^54^, ^55^ These findings underscore the broader impact of ECPR beyond individual survival outcomes and highlight the importance of incorporating organ donation into cost-effectiveness analyses.^48^

However, another important knowledge gap remains: the differential impact of prehospital versus in-hospital ECPR on organ donation outcomes. Given that prehospital ECPR may expand the pool of eligible candidates by reducing low-flow times,^17^ its potential effect on organ donation and transplantation outcomes warrants further investigation. Understanding these dynamics is crucial to fully assess the performance and cost-effectiveness of different ECPR deployment models and to inform policy decisions regarding their implementation.

The ongoing ON-SCENE trial (NCT04620070),^18^ a non-randomized stepped wedge trial comparing deployment of HEMS not equipped with ECPR with HEMS equipped with ECPR, will shed light on the effectiveness of prehospital ECPR compared to standard ACLS protocols. However, it will not address the comparative efficacy of prehospital ECPR versus in-hospital ECPR. To address this gap, future studies should focus on optimizing ECPR integration within the ‘chain of survival’ rather than solely contrasting it with ACLS. Given healthcare system variability, prospective trials comparing prehospital and in-hospital ECPR in terms of survival with favorable neurological outcomes are needed in multiple settings to individualize implementation decisions.

## Conclusion

This systematic review identified eight single-arm studies of prehospital ECPR, reporting a pooled survival with good neurological function of 25% and a pooled low-flow time of 58 min. Across studies, shorter low-flow time appeared to be associated with improved outcomes. Three comparative studies showed a consistent reduction in low-flow time of approximately 30 min with prehospital ECPR and a non-significant trend toward improved survival.

## Declaration of AI and AI-assisted technologies in the writing process

During the preparation of this work the author(s) used ChatGPT to review syntax and orthograph to improve readability and language. After using this tool/service, the author(s) reviewed and edited the content as needed and take(s) full responsibility for the content of the publication.

## CRediT authorship contribution statement

**Lawrence Leroux:** Conceptualization, Data curation, Formal analysis, Investigation, Methodology, Project administration, Software, Validation, Writing – original draft, Writing – review & editing. **Nathaniel B. Dennis-Benford:** Investigation, Writing – review & editing. **Amy Bergeron:** Methodology, Resources, Writing – original draft, Writing – review & editing. **Lionel Lamhaut:** Writing – review & editing. **Alexis Cournoyer:** Writing – review & editing. **Brian Grunau:** Writing – review & editing. **Yiorgos Alexandros Cavayas:** Conceptualization, Methodology, Supervision, Writing – review & editing.

## Declaration of competing interest

The authors declare that they have no known competing financial interests or personal relationships that could have appeared to influence the work reported in this paper.

## Figures and Tables

**Table:** 

**#**	**Search**	**Results**
1	(pre?hospital* or pre-hospital* or extra?hospital* or extra-hospital* or mobile or portable or transit* or transport* or transfer* or helicopter* or airborn* or aircraft* or airliner* or airplane* or aeroplane* or plane* or ambulance* or ambulatory or emergency medical technician* or emt? or field* or first respon* or disaster*).tw,kf.	3,549,128
2	(((extracorporeal* or venoarterial*) adj2 membrane* adj2 oxygen*) or ECMO).tw,kf.	21,858
3	((extracorporeal* adj2 resuscitat*) or ECPR).tw,kf.	1545
4	2 or 3	22,526
5	((pre?hospital* or pre-hospital* or extra?hospital* or extra-hospital* or mobile or portable or transit* or transport* or transfer* or helicopter* or airborn* or aircraft* or airliner* or airplane* or aeroplane* or plane* or ambulance* or ambulatory or emergency medical technician* or emt? or field* or first respon* or disaster*) adj5 (((extracorporeal* or venoarterial*) adj2 membrane* adj2 oxygen*) or ECMO or ((extracorporeal* adj2 resuscitat*) or ECPR))).tw,kf. [prehospital ecmo]	800
6	cannulat*.tw,kf.	29,095
7	((pre?hospital* or pre-hospital* or extra?hospital* or extra-hospital* or mobile or portable or transit* or transport* or transfer* or helicopter* or airborn* or aircraft* or airliner* or airplane* or aeroplane* or plane* or ambulance* or ambulatory or emergency medical technician* or emt? or field* or first respon* or disaster*) adj5 cannulat*).tw,kf.	217
8	4 and 7 [prehospital ecmo − ooh cannulation & ecmo]	62
9	5 or 8 [prehospital ecmo master line]	817
10	Out-of-Hospital Cardiac Arrest/	7726
11	OHCA.tw,kf.	4842
12	(out-of-hospital* or ((out or outside) adj2 hospital*) or out-hospital* or pre?hospital* or pre-hospital* or extra?hospital* or extra-hospital* or at-home or workplace* or drown* or helicopter* or airborn* or aircraft* or airliner* or airplane* or aeroplane* or plane*).tw,kf.	400,916
13	(arrest* or CA or heart attack* or pulseless*).tw,kf.	496,538
14	((out-of-hospital* or ((out or outside) adj2 hospital*) or out-hospital* or pre?hospital* or pre-hospital* or extra?hospital* or extra-hospital* or at-home or workplace* or drown* or helicopter* or airborn* or aircraft* or airliner* or airplane* or aeroplane* or plane*) adj6 (arrest* or CA or heart attack* or pulseless*)).tw,kf. [ohca, explicit, keywording]	11,418
15	(prehospital or pre-hospital).jw.	5365
16	13 and 15 [ohca, implicit − “prehospital” journals]	601
17	Heart Arrest/ or exp Death, Sudden, Cardiac/	51,093
18	Emergency Medical Technicians/ or Ambulances/ or Disaster Medicine/ or Emergency Medical Services/ or Rescue Work/	60,272
19	17 and 18 [ohca, implicit − heart arrest + emt, ambulance or disaster, MeSH]	2939
20	(ambulance* or emergency medical technician* or emt? or field* or first respon* or disaster* or helicopter* or aircraft*).tw,kf.	1,290,334
21	((ambulance* or emergency medical technician* or emt? or field* or first respon* or disaster* or helicopter* or aircraft*) adj6 (arrest* or CA or heart attack* or pulseless*)).tw,kf. [ohca, implicit − heart arrest + emt, ambuldance or disaster, keywording]	2032
22	exp Heart Failure/ or exp Heart Rupture/	158,522
23	exp Myocardial Infarction/	198,376
24	22 or 23	342,818
25	12 or 15 or 20 [ooh indicators, implicit and explicit]	1,640,103
26	24 and 25 [ohca, implicit − heart failure/rupture or MI, MeSH, + ooh indicators, implicit or explicit, kw]	9012
27	(myocardial infarct* or minoca or cardiogenic shock).tw,kf.	247,453
28	((heart or cardio* or cardiac* or ventricul*) adj3 (fail* or ruptur*)).tw,kf.	264,275
29	27 or 28	473,977
30	((out-of-hospital* or ((out or outside) adj2 hospital*) or out-hospital* or pre?hospital* or pre-hospital* or extra?hospital* or extra-hospital* or at-home or workplace* or drown* or helicopter* or airborn* or aircraft* or airliner* or airplane* or aeroplane* or plane*) adj6 (myocardial infarct* or minoca or cardiogenic shock or ((heart or cardio* or cardiac* or ventricul*) adj3 (fail* or ruptur*)))).tw,kf. [ohca, implicit − heart failure/rupture or MI, kw, + ooh indicators, implicit or explicit, kw]	1415
31	10 or 11 or 14 or 16 or 19 or 21 or 26 or 30 [ohca master line]	25,317
32	9 and 31 [prehospital ecmo for ohca]	116
33	((newborn* or new-born* or neonat* or neo-nat* or infan* or child* or adolesc* or p?ediatr* or baby* or babies* or toddler* or kid or kids or boy* or girl* or juvenile* or teen* or youth* or pubescen* or preadolesc* or prepubesc* or preteen or tween) not adult*).ti,kf,jw.	2,250,433
34	32 not 33 [results, adult only]	109

**Table:** 

**#**	**Search**	**Results**
1	(pre?hospital* or pre-hospital* or extra?hospital* or extra-hospital* or mobile or portable or transit* or transport* or transfer* or helicopter* or airborn* or aircraft* or airliner* or airplane* or aeroplane* or plane* or ambulance* or ambulatory or emergency medical technician* or emt? or field* or first respon* or disaster*).tw,kf.	4,085,825
2	(((extracorporeal* or venoarterial*) adj2 membrane* adj2 oxygen*) or ECMO).tw,kf.	38,886
3	((extracorporeal* adj2 resuscitat*) or ECPR).tw,kf.	2586
4	2 or 3	39,873
5	((pre?hospital* or pre-hospital* or extra?hospital* or extra-hospital* or mobile or portable or transit* or transport* or transfer* or helicopter* or airborn* or aircraft* or airliner* or airplane* or aeroplane* or plane* or ambulance* or ambulatory or emergency medical technician* or emt? or field* or first respon* or disaster*) adj5 (((extracorporeal* or venoarterial*) adj2 membrane* adj2 oxygen*) or ECMO or ((extracorporeal* adj2 resuscitat*) or ECPR))).tw,kf. [prehospital ecmo]	1737
6	cannulat*.tw,kf.	43,840
7	((pre?hospital* or pre-hospital* or extra?hospital* or extra-hospital* or mobile or portable or transit* or transport* or transfer* or helicopter* or airborn* or aircraft* or airliner* or airplane* or aeroplane* or plane* or ambulance* or ambulatory or emergency medical technician* or emt? or field* or first respon* or disaster*) adj5 cannulat*).tw,kf.	384
8	4 and 7 [prehospital ecmo − ooh cannulation & ecmo]	164
9	5 or 8 [prehospital ecmo master line]	1785
10	out of hospital cardiac arrest/	16,157
11	OHCA.tw,kf.	8848
12	(out-of-hospital* or ((out or outside) adj2 hospital*) or out-hospital* or pre?hospital* or pre-hospital* or extra?hospital* or extra-hospital* or at-home or workplace* or drown* or helicopter* or airborn* or aircraft* or airliner* or airplane* or aeroplane* or plane*).tw,kf.	486,765
13	(arrest* or CA or heart attack* or pulseless*).tw,kf.	761,244
14	((out-of-hospital* or ((out or outside) adj2 hospital*) or out-hospital* or pre?hospital* or pre-hospital* or extra?hospital* or extra-hospital* or at-home or workplace* or drown* or helicopter* or airborn* or aircraft* or airliner* or airplane* or aeroplane* or plane*) adj6 (arrest* or CA or heart attack* or pulseless*)).tw,kf. [ohca, explicit, keywording]	18,737
15	(prehospital or pre-hospital).jx.	5451
16	13 and 15 [ohca, implicit − “prehospital” journals]	615
17	heart arrest/ or exp sudden cardiac death/	116,391
18	rescue personnel/ or exp ambulance/ or disaster medicine/ or emergency health service/ or disaster response/ or rescue work/	140,096
19	17 and 18 [ohca, implicit − heart arrest + emt, ambulance or disaster, MeSH]	5416
20	(ambulance* or emergency medical technician* or emt? or field* or first respon* or disaster* or helicopter* or aircraft*).tw,kf.	1,429,496
21	((ambulance* or emergency medical technician* or emt? or field* or first respon* or disaster* or helicopter* or aircraft*) adj6 (arrest* or CA or heart attack* or pulseless*)).tw,kf. [ohca, implicit − heart arrest + emt, ambuldance or disaster, keywording]	2945
22	exp heart failure/ or heart rupture/	684,132
23	exp heart infarction/	465,705
24	22 or 23	1,036,920
25	12 or 15 or 20 [ooh indicators, implicit and explicit]	1,857,941
26	24 and 25 [ohca, implicit − heart failure/rupture or MI, MeSH, + ooh indicators, implicit or explicit, kw]	51,022
27	(myocardial infarct* or minoca or cardiogenic shock).tw,kf.	364,146
28	((heart or cardio* or cardiac* or ventricul*) adj3 (fail* or ruptur*)).tw,kf.	432,895
29	27 or 28	731,121
30	((out-of-hospital* or ((out or outside) adj2 hospital*) or out-hospital* or pre?hospital* or pre-hospital* or extra?hospital* or extra-hospital* or at-home or workplace* or drown* or helicopter* or airborn* or aircraft* or airliner* or airplane* or aeroplane* or plane*) adj6 (myocardial infarct* or minoca or cardiogenic shock or ((heart or cardio* or cardiac* or ventricul*) adj3 (fail* or ruptur*)))).tw,kf. [ohca, implicit − heart failure/rupture or MI, kw, + ooh indicators, implicit or explicit, kw]	2214
31	10 or 11 or 14 or 16 or 19 or 21 or 26 or 30 [ohca master line]	59,471
32	9 and 31 [prehospital ecmo for ohca]	285
33	((newborn* or new-born* or neonat* or neo-nat* or infan* or child* or adolesc* or p?ediatr* or baby* or babies* or toddler* or kid or kids or boy* or girl* or juvenile* or teen* or youth* or pubescen* or preadolesc* or prepubesc* or preteen or tween) not adult*).ti,kf,jx.	2,648,529
34	32 not 33 [results, adult only]	256
35	limit 34 to (conference abstracts or conference abstract status or conference abstract or “conference review” or conference proceeding)	116
36	limit 35 to yr=“2022 −Current”	37
37	34 not 35	140
38	36 or 37	177

**Table:** 

**#**	**Question**	**Résultats**
S33	S31 NOT S32	45
S32	TI ((newborn* or new-born* or neonat* or neo-nat* or infan* or child* or adolesc* or p#ediatr* or baby* or babies* or toddler* or kid or kids or boy* or girl* or juvenile* or teen* or youth* or pubescen* or preadolesc* or prepubesc* or preteen or tween) not adult*) OR SO ((newborn* or new-born* or neonat* or neo-nat* or infan* or child* or adolesc* or p#ediatr* or baby* or babies* or toddler* or kid or kids or boy* or girl* or juvenile* or teen* or youth* or pubescen* or preadolesc* or prepubesc* or preteen or tween) not adult*)	889,529
S31	S9 AND S30	48
S30	S10 OR S13 OR S15 OR S18 OR S20 OR S25 OR S29	12,809
S29	TI ((out-of-hospital* or ((out or outside) N1 hospital*) or out-hospital* or pre#hospital* or pre-hospital* or extra#hospital* or extra-hospital* or at-home or workplace* or drown* or helicopter* or airborn* or aircraft* or airliner* or airplane* or aeroplane* or plane*) N5 (myocardial infarct* or minoca or cardiogenic shock or ((heart or cardio* or cardiac* or ventricul*) N2 (fail* or ruptur*)))) OR AB ((out-of-hospital* or ((out or outside) N1 hospital*) or out-hospital* or pre#hospital* or pre-hospital* or extra#hospital* or extra-hospital* or at-home or workplace* or drown* or helicopter* or airborn* or aircraft* or airliner* or airplane* or aeroplane* or plane*) N5 (myocardial infarct* or minoca or cardiogenic shock or ((heart or cardio* or cardiac* or ventricul*) N2 (fail* or ruptur*))))	1,058
S28	S26 OR S27	120,749
S27	TI ((heart or cardio* or cardiac* or ventricul*) N2 (fail* or ruptur*)) OR AB ((heart or cardio* or cardiac* or ventricul*) N2 (fail* or ruptur*))	72,012
S26	TI (myocardial infarct* or minoca or cardiogenic shock) OR AB (myocardial infarct* or minoca or cardiogenic shock)	56,485
S25	S23 AND S24	4,389
S24	S11 OR S14 OR S19	419,315
S23	S21 OR S22	96,848
S22	(MH “Myocardial Infarction+”)	50,123
S21	(MH “Heart Failure+”) or (MH “Heart Rupture+”)	50,636
S20	TI ((ambulance* or emergency medical technician* or emt# or field* or first respon* or disaster* or helicopter* or aircraft*) N5 (arrest* or“CA” or heart attack* or pulseless*)) OR AB ((ambulance* or emergency medical technician* or emt# or field* or first respon* or disaster* or helicopter* or aircraft*) N5 (arrest* or“CA” or heart attack* or pulseless*))	386
S19	TI (ambulance* or emergency medical technician* or emt# or field* or first respon* or disaster* or helicopter* or aircraft*) OR AB (ambulance* or emergency medical technician* or emt# or field* or first respon* or disaster* or helicopter* or aircraft*)	171,014
S18	S16 AND S17	2,367
S17	(MH “Emergency Medical Technicians”) or (MH “Ambulances”) or (MH “Emergency Medical Services”) OR (MH “Rescue Work+”)	41,678
S16	(MH “Heart Arrest+”)	23,213
S15	S12 AND S14	571
S14	SO (prehospital or pre-hospital)	6,696
S13	TI ((out-of-hospital* or ((out or outside) N1 hospital*) or out-hospital* or pre#hospital* or pre-hospital* or extra#hospital* or extra-hospital* or at-home or workplace* or drown* or helicopter* or airborn* or aircraft* or airliner* or airplane* or aeroplane* or plane*) N5 (arrest* or “CA” or heart attack* or pulseless*)) OR AB ((out-of-hospital* or ((out or outside) N1 hospital*) or out-hospital* or pre#hospital* or pre-hospital* or extra#hospital* or extra-hospital* or at-home or workplace* or drown* or helicopter* or airborn* or aircraft* or airliner* or airplane* or aeroplane* or plane*) N5 (arrest* or “CA” or heart attack* or pulseless*))	6,495
S12	TI (arrest* or “CA” or heart attack* or pulseless*) OR AB (arrest* or “CA” or heart attack* or pulseless*)	57,424
S11	TI (out-of-hospital* or ((out or outside) N1 hospital*) or out-hospital* or pre#hospital* or pre-hospital* or extra#hospital* or extra-hospital* or at-home or workplace* or drown* or helicopter* or airborn* or aircraft* or airliner* or airplane* or aeroplane* or plane*) OR AB (out-of-hospital* or ((out or outside) N1 hospital*) or out-hospital* or pre#hospital* or pre-hospital* or extra#hospital* or extra-hospital* or at-home or workplace* or drown* or helicopter* or airborn* or aircraft* or airliner* or airplane* or aeroplane* or plane*)	260,642
S10	TI OHCA OR AB OHCA	2,304
S9	S5 or S8	299
S8	S4 and S7	21
S7	TI ((pre#hospital* or pre-hospital* or extra#hospital* or extra-hospital* or mobile or portable or transit* or transport* or transfer* or helicopter* or airborn* or aircraft* or airliner* or airplane* or aeroplane* or plane* or ambulance* or ambulatory or emergency medical technician* or emt# or field* or first respon* or disaster*) N4 cannulat*) OR AB ((pre#hospital* or pre-hospital* or extra#hospital* or extra-hospital* or mobile or portable or transit* or transport* or transfer* or helicopter* or airborn* or aircraft* or airliner* or airplane* or aeroplane* or plane* or ambulance* or ambulatory or emergency medical technician* or emt# or field* or first respon* or disaster*) N4 cannulat*)	45
S6	TI cannulat* OR AB cannulat*	5,46
S5	TI ((pre#hospital* or pre-hospital* or extra#hospital* or extra-hospital* or mobile or portable or transit* or transport* or transfer* or helicopter* or airborn* or aircraft* or airliner* or airplane* or aeroplane* or plane* or ambulance* or ambulatory or emergency medical technician* or emt# or field* or first respon* or disaster*) N4 (((extracorporeal* or venoarterial*) N1 membrane* N1 oxygen*) or ECMO or ((extracorporeal* N1 resuscitat*) or ECPR))) OR AB ((pre#hospital* or pre-hospital* or extra#hospital* or extra-hospital* or mobile or portable or transit* or transport* or transfer* or helicopter* or airborn* or aircraft* or airliner* or airplane* or aeroplane* or plane* or ambulance* or ambulatory or emergency medical technician* or emt# or field* or first respon* or disaster*) N4 (((extracorporeal* or venoarterial*) N1 membrane* N1 oxygen*) or ECMO or ((extracorporeal* N1 resuscitat*) or ECPR)))	295
S4	S2 or S3	7,946
S3	TI ((extracorporeal* N1 resuscitat*) or ECPR) OR AB ((extracorporeal* N1 resuscitat*) or ECPR)	658
S2	TI (((extracorporeal* or venoarterial*) N1 membrane* N1 oxygen*) or ECMO) OR AB (((extracorporeal* or venoarterial*) N1 membrane* N1 oxygen*) or ECMO)	7,509
S1	TI (pre#hospital* or pre-hospital* or extra#hospital* or extra-hospital* or mobile or portable or transit* or transport* or transfer* or helicopter* or airborn* or aircraft* or airliner* or airplane* or aeroplane* or plane* or ambulance* or ambulatory or emergency medical technician* or emt# or field* or first respon* or disaster*) OR AB (pre#hospital* or pre-hospital* or extra#hospital* or extra-hospital* or mobile or portable or transit* or transport* or transfer* or helicopter* or airborn* or aircraft* or airliner* or airplane* or aeroplane* or plane* or ambulance* or ambulatory or emergency medical technician* or emt# or field* or first respon* or disaster*)	431,534

**Table:** 

**#**	**Search Query**	**Results**
1	TS=(pre$hospital* or pre-hospital* or extra$hospital* or extra-hospital* or mobile or portable or transit* or transport* or transfer* or helicopter* or airborn* or aircraft* or airliner* or airplane* or aeroplane* or plane* or ambulance* or ambulatory or (emergency NEAR/0 medical NEAR/0 technician*) or emt$ or field* or (first NEAR/0 respon*) or disaster*)	11,906,569
2	TS=(((extracorporeal* or venoarterial*) NEAR/1 membrane* NEAR/1 oxygen*) or ECMO)	26,608
3	TS=((extracorporeal* NEAR/1 resuscitat*) or ECPR)	1826
4	#2 or #3	27,526
5	TS=((pre$hospital* or pre-hospital* or extra$hospital* or extra-hospital* or mobile or portable or transit* or transport* or transfer* or helicopter* or airborn* or aircraft* or airliner* or airplane* or aeroplane* or plane* or ambulance* or ambulatory or (emergency NEAR/0 medical NEAR/0 technician*) or emt$ or field* or (first NEAR/0 respon*) or disaster*) NEAR/4 (ECMO or ECPR or (extracorporeal* NEAR/1 membrane* NEAR/1 oxygen*) or (venoarterial* NEAR/1 membrane* NEAR/1 oxygen*) or extracorporeal* NEAR/1 resuscitat*))	743
6	TS = cannulat*	30,530
7	TS=((pre$hospital* or pre-hospital* or extra$hospital* or extra-hospital* or mobile or portable or transit* or transport* or transfer* or helicopter* or airborn* or aircraft* or airliner* or airplane* or aeroplane* or plane* or ambulance* or ambulatory or (emergency NEAR/0 medical NEAR/0 technician*) or emt$ or field* or (first NEAR/0 respon*) or disaster*) NEAR/4 cannulat*)	207
8	#4 and #7	61
9	#5 or #8	759
10	TS=OHCA	4726
11	TS=(out-of-hospital* or ((out or outside) NEAR/1 hospital*) or out-hospital* or pre$hospital* or pre-hospital* or extra$hospital* or extra-hospital* or at-home or workplace* or drown* or helicopter* or airborn* or aircraft* or airliner* or airplane* or aeroplane* or plane*)	1,421,363
12	TS=(arrest* or CA or (heart NEAR/0 attack*) or pulseless*)	863,220
13	TS=((out-of-hospital* or ((out or outside) NEAR/1 hospital*) or out-hospital* or pre$hospital* or pre-hospital* or extra$hospital* or extra-hospital* or at-home or workplace* or drown* or helicopter* or airborn* or aircraft* or airliner* or airplane* or aeroplane* or plane*) NEAR/5 (arrest* or CA or (heart NEAR/0 attack*) or pulseless*))	13,565
14	SO = (“PREHOSPITAL AND DISASTER MEDICINE” OR PREHOSPITAL EMERGENCY CARE)	3613
15	#12 and #14	567
16	TS=(ambulance* or (emergency NEAR/0 medical NEAR/0 technician*) or emt$ or field* or (first NEAR/0 respon*) or disaster* or helicopter* or aircraft*)	5,069,748
17	TS=((ambulance* or (emergency NEAR/0 medical NEAR/0 technician*) or emt$ or field* or (first NEAR/0 respon*) or disaster* or helicopter* or aircraft*) NEAR/5 (arrest* or CA or (heart NEAR/0 attack*) or pulseless*))	3963
18	TS=((myocardial NEAR/0 infarct*) or minoca or (cardiogenic NEAR/0 shock))	357,734
19	TS=((heart or cardio* or cardiac* or ventricul*) NEAR/2 (fail* or ruptur*))	354,702
20	#18 or #19	653,935
21	TS=((out-of-hospital* or ((out or outside) NEAR/1 hospital*) or out-hospital* or pre$hospital* or pre-hospital* or extra$hospital* or extra-hospital* or at-home or workplace* or drown* or helicopter* or airborn* or aircraft* or airliner* or airplane* or aeroplane* or plane*) NEAR/5 ((myocardial NEAR/0 infarct*) or minoca or (cardiogenic NEAR/0 shock) or ((heart or cardio* or cardiac* or ventricul*) NEAR/2 (fail* or ruptur*))))	1348
22	#10 or #11 or #13 or #15 or #17 or #21	1,424,978
23	#9 and #22	181
24	TI=((newborn* or new-born* or neonat* or neo-nat* or infan* or child* or adolesc* or p$ediatr* or baby* or babies* or toddler* or kid or kids or boy* or girl* or juvenile* or teen* or youth* or pubescen* or preadolesc* or prepubesc* or preteen or tween) not adult*) or SO=((newborn* or new-born* or neonat* or neo-nat* or infan* or child* or adolesc* or p$ediatr* or baby* or babies* or toddler* or kid or kids or boy* or girl* or juvenile* or teen* or youth* or pubescen* or preadolesc* or prepubesc* or preteen or tween) not adult*)	2,269,376
25	#23 not #24	170

**Table:** 

Criteria
1. Was the study question or objective clearly stated?
2. Was the study population clearly and fully described, including a case definition?
3. Were the cases consecutive?
4. Were the subjects comparable?
5. Was the intervention clearly described?
6. Were the outcome measures clearly defined, valid, reliable, and implemented consistently across all study participants?
7. Was the length of follow-up adequate?
8. Were the statistical methods well-described?
9. Were the results well-described?

**Table:** 

## Appendix A

Research Strategies

The strategies for all sources and search dates have been previously reported in a dataset published on Boréalis with a Creative Commons CC-BY license: https://doi.org/10.5683/SP3/6OAEWB.

Medline ALL (Ovid)

Ovid MEDLINE(R) ALL < 1946 to July 02, 2024>Embase (Ovid)

Embase < 1974 to 2024 July 02>CINAHL Complete (EBSCOhost)Web of Science (Clarivate)

Segments: WOS.SCI: 1945 to 2024, WOS.AHCI: 1975 to 2024, WOS.ESCI: 2019 to 2024, WOS.ISTP: 1990 to 2024, WOS.SSCI: 1956 to 2024. WOS.ISSHP: 1990 to 2024

## Appendix B

NIH Quality Assessment Tool for Case Series Studies.
